# Simultaneous assessment of stress hyperglycemia ratio and glucose variability to predict all-cause mortality in sepsis patients across different glucose metabolic states: an observational cohort study with interpretable machine learning approach

**DOI:** 10.1097/JS9.0000000000003525

**Published:** 2025-09-23

**Authors:** Fuxu Wang, Yu Guo, Chucheng Jiao, Shuangmei Zhao, Liutao Sui, Zhi Mao, Ruogu Lu, Rongyao Hou, Xiaoyan Zhu

**Affiliations:** a Department of Critical Care Medicine, The Affiliated Hospital of Qingdao University, Qingdao, China; b Department of Neurology, The Affiliated Hospital of Qingdao University, Qingdao, China; c Department of Critical Care Medicine, The First Medical Center of PLA General Hospital, Beijing, China; d Medical Innovation Research Department, Chinese PLA General Hospital, Beijing, China; e Department of Neurology, The Affiliated Hiser Hospital of Qingdao University, Qingdao, China

**Keywords:** glycemic variability, machine learning, MIMIC-IV database, sepsis, stress hyperglycemia ratio

## Abstract

**Background:**

Stress hyperglycemia ratio (SHR) and glycemic variability (GV) reflect acute glucose elevation and fluctuation, which are associated with adverse outcomes in patients with some diseases. However, the relationship between combined assessment of SHR and GV and mortality risk in sepsis remains unclear. This study aims to investigate the associations of SHR, GV, and their combination with sepsis mortality among individuals with different glucose metabolic states, and to develop a mortality prediction model using machine learning (ML) models.

**Methods:**

Patients with sepsis were screened in the MIMIC-IV database, stratified into normal glucose regulation (NGR), prediabetes mellitus (Pre-DM), and diabetes mellitus (DM) groups based on glucose metabolic status. Associations with mortality were analyzed using Kaplan–Meier (KM) curves, Cox proportional hazards model, restricted cubic splines (RCS), and landmark analyses. Five ML algorithms were employed for prediction, with SHapley Additive explanations (SHAP) interpreting key predictors.

**Results:**

A total of 4838 patients were enrolled, with a median age of 68 years. Overall, 641 patients (13.2%) died in the ICU, and 936 patients (19.3%) died within 28 days after admission to the ICU. In NGR patients, combined high SHR (>1.23; highest tertile) and high GV (>28.56; highest tertile) – determined based on tertile distribution – conferred the highest 28-day mortality risk (HR = 2.06, 95% CI: 1.40–3.04). Pre-DM patients with low SHR/high GV (SHR < 1.23, GV > 28.56) showed the greatest 28-day mortality risk (HR = 2.45, 95% CI: 1.73–3.48). DM patients with high SHR/low GV (SHR > 1.23, GV < 28.56) had the highest 28-day mortality risk (HR = 1.46, 95% CI: 1.06–2.01). Machine learning models – particularly XGBoost (AUC: 0.746), Random Forest (AUC: 0.776), and Logistic Regression (AUC: 0.776) – demonstrated the strongest predictive performance for these endpoints.

**Conclusion:**

The combined assessment of SHR and GV may provide useful information for predicting mortality in sepsis patients – particularly among individuals with NGR and Pre-DM. This integrated approach highlights the potential need for personalized glycemic management strategies, which warrants further investigation in prospective studies.

**HIGHLIGHTS**

- The study revealed that integrating SHR and GV enhances mortality prediction in sepsis, particularly for patients with normal glucose regulation (NGR) and prediabetes, thereby advocating for personalized glycemic management strategies tailored to metabolic phenotypes.
- Machine learning models incorporating SHR and GV demonstrated robust prognostic value, suggesting their potential to inform clinical decision-making and risk stratification in sepsis care.

## Introduction

Sepsis, a life-threatening organ dysfunction caused by a dysregulated host response to infection, remains a leading cause of mortality in intensive care units^[1,2]^. Dysglycemia, a hallmark of metabolic disturbance in sepsis, worsens outcomes through oxidative stress, endothelial dysfunction, and immune suppression[3]. However, the impact of hyperglycemia on mortality varies across patients with different glucose metabolic statuses[4], underscoring the need for refined glycemic evaluation metrics.

Stress hyperglycemia, marked by elevated admission blood glucose (ABG) due to stressors, arises via sympathetic activation, inflammatory cytokine release, and hypothalamic-pituitary-adrenal axis hyperactivation^[5,6]^. Since ABG may be confounded by chronic glycemia, the stress hyperglycemia ratio (SHR) adjusts for baseline glycemic status to quantify true acute disturbances^[7,8]^, showing stronger prognostic value in nondiabetic ICU patients[9]. Glycemic variability (GV), the degree of glucose fluctuation over time[10], is linked to adverse outcomes in heart failure, traumatic brain injury, cardiovascular disease, and diabetes^[6,11–13]^, often outperforming isolated hyperglycemia as a mortality predictor[14]. Combined SHR and GV assessment may thus enhance risk stratification and guide glycemic management in critical care.

Current evidence suggests differential mortality implications of hyperglycemia between diabetic and nondiabetic populations, with some studies indicating attenuated clinical risks associated with acute hyperglycemia in diabetic ICU patients – a phenomenon potentially attributable to chronic metabolic adaptation mechanisms^[4,15]^. Although previous studies have reported the prognostic value of SHR and GV in critically ill patients, most did not systematically stratify by glucose metabolic status, lacked evaluation of the time-dependent effects of combined SHR and GV, and rarely integrated interpretable machine learning approaches with traditional regression models in the sepsis population. We hypothesize that the combination of SHR and GV is independently associated with an increased risk of mortality in sepsis, and that this risk varies among sepsis patients with different glucose metabolic statuses.

To test this hypothesis, the present study utilized a large MIMIC-IV sepsis cohort to comprehensively assess the independent and joint effects of SHR and GV across three metabolic phenotypes (NGR, Pre-DM, and DM), apply landmark analysis to identify temporal heterogeneity in prognostic impact, and incorporate Boruta feature selection with SHAP interpretability to enhance model transparency.

## Methods

### Data source

This retrospective cohort study utilized data from the Medical Information Mart for Intensive Care-IV (MIMIC-IV) database (version 3.1), a publicly accessible repository containing de-identified clinical records of >70 000 ICU admissions at Beth Israel Deaconess Medical Center (2008–2019)[16]. The dataset encompasses multidimensional variables, including demographic characteristics, longitudinal vital signs, laboratory biomarkers, fluid balance dynamics, and survival outcomes.

### Ethical approval

This retrospective study was conducted using the publicly accessible MIMIC-IV database, which comprises routinely collected clinical data that were fully de-identified before release, with no direct patient identifiers. According to the database’s official statement, access is permitted for research under strict data use agreements, and informed consent is not required due to the anonymized nature of the data. All analyses complied with the Health Insurance Portability and Accountability Act (HIPAA) standards and the ethical principles of the Declaration of Helsinki. The study adheres to the STROBE (Strengthening the Reporting of Observational Studies in Epidemiology) guidelines and has been reported in line with the STROCSS (Strengthening the Reporting of Cohort Studies in Surgery) criteria[17]. Given these safeguards, additional prospective registration and individual informed consent were not necessary under institutional and national regulations.

### Study population

**Figure 1.**
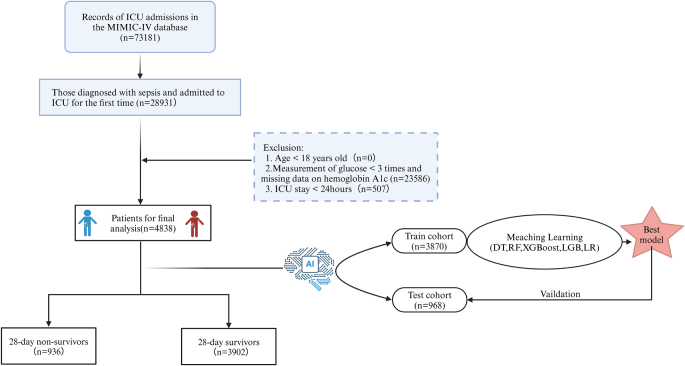
Flow chart of the study design.

ICU, intensive care unit; LR, logistic regression; DT, decision tree; RF, random forest; XGBoost, extreme gradient boosting; LGB, light gradient boosting machine.

A detailed data extraction process was developed, with pilot extractions performed before the formal phase to evaluate and refine the clarity and feasibility of the aforementioned procedures. To ensure data accuracy, multiple validation methods were implemented, including independent verification of critical data points and statistical software-assisted consistency checks to identify and rectify potential input errors or discrepancies. Included patients diagnosed with sepsis according to the recommendations of the Third International Consensus Definitions for Sepsis and Septic Shock (Sepsis-3). Exclusion criteria comprised: (1) age <18 years, (2) ICU stay duration <24 hours, and (3) fewer than three blood glucose measurements or missing HbA1c data during the index ICU admission[18]. For patients with multiple ICU admissions, only data from the first admission were retained. After exclusions, 4834 patients were included in the final cohort extracted from the MIMIC-IV database. The flowchart of the patient inclusion process is shown in Figure 1.

### Data extraction

The data was extracted using the Structured Query Language (SQL) with Navicat Premium (Version 16.3.11). The process targeted five main areas of data acquisition: (1) demographic data, including age, sex, height, weight, and ethnic background. (2) Clinical severity indices, including the Glasgow Coma Scale (GCS), Sequential Organ Failure Assessment (SOFA) score, and Charlson score. (3) Vital signs, including arterial blood pressure-systolic (Abps), arterial blood pressure-diastolic (Abpd), heart rate (HR), body temperature in degrees Celsius. (4) Haematological and biochemical parameters, including haemoglobin concentration (Hb), red blood cell count (RBC), platelet (Plt) count, white blood cell count (WBC), albumin, alanine aminotransferase (ALT), aspartate aminotransferase (AST), blood urea nitrogen (BUN), Glucose, HbA1c, PH, Lactate, and Creatinine. (5) Existing comorbidities such as hypertension, Type 2 diabetes(T2DM), heart failure (HF), chronic obstructive pulmonary disease (COPD), acute kidney injury (AKI), hyperlipidemia (HLD), Hypertension (HTN), and therapeutic interventions, including the use of mechanical ventilation, hypoglycemic drugs, and Insulin(RI). SHR was calculated by the following equation: [plasma glucose (mg/dL)/(28.7 × HbA1c (%) − 46.7)][19]. GV was expressed as the coefficient of variation, defined as the ratio of the standard deviation to the mean of all repeat plasma glucose measurements during the ICU stay[20]. Body mass index (BMI) = weight (kg)/height^2^ (m^2^).

All laboratory variables were first measured after ICU admission. Missing data for continuous variables were imputed using multiple imputation by chained equations (MICE) with five iterations, assuming missing at random (MAR). For categorical variables, probabilistic imputation was applied based on observed category distributions. Variables with more than 20% missingness were excluded from the analysis.

### Grouping of the study

We performed phenotype-stratified risk assessment by simultaneously evaluating SHR and GV across three distinct glucose metabolic states – normal glucose regulation (NGR), prediabetes mellitus (Pre-DM), and diabetes mellitus (DM). The NGR group consisted of individuals with a glycated hemoglobin (HbA1c) level below 5.7% and no prior history of diabetes. Those falling into the pre-DM category had an HbA1c level ranging from 5.7% (inclusive) to 6.5% and no previous diabetes history. As for the DM group, it included patients who either had a history of diabetes or an HbA1c level ≥6.5%[21].

### Establishment and validation of the prediction models

The dataset was randomly partitioned into training (80%) and testing (20%) cohorts. Boruta algorithm-selected predictors were used to develop five machine learning (ML) models: logistic regression (LR), decision tree (DT), random forest (RF), extreme gradient boosting (XGBoost), and light gradient boosting machine (LightGBM). A baseline model was first trained without hyperparameter tuning, and performance was evaluated on the independent test set. Subsequently, hyperparameter optimization was performed using a grid search across a parameter grid. The grid search used 5-fold cross-validation on the training dataset, with the area under the receiver operating characteristic curve (AUC) as the scoring metric. The optimal model was then retrained on the entire training set and evaluated on the test set. Model performance was assessed using AUC, sensitivity, specificity, accuracy, and F1-score. Sensitivity and specificity were calculated at the optimal threshold determined by maximizing the Youden index from the ROC curve. SHapley additive explanations (SHAP) explored the interpretability of the final prediction model[22].

### AI/ML transparency statement

Purpose: ML models were applied to evaluate the prognostic significance of SHR, GV, and other clinical variables in predicting 28-day mortality among septic patients.

Tool Specifications: Implemented in Python 3.9 using scikit-learn (LogisticRegression, DecisionTreeClassifier, RandomForestClassifier, GradientBoosting), BorutaPy, XGBoost, LightGBM, and SHAP (LinearExplainer).

Data inputs and preprocessing: Continuous variables were imputed using MICE (five iterations, MAR assumption); categorical variables via probabilistic imputation; features were standardized. Variables with >20% missingness were excluded.

Human oversight: All algorithm choices, parameter ranges, and interpretability assessments were overseen by the research team with clinical and statistical expertise.

Bias mitigation: Class imbalance was addressed during model training and stratified cross-validation folds.class_weight = “balanced.”

Reproducibility: Random seeds were fixed; all preprocessing, parameter settings, and analysis scripts are archived and available upon reasonable request.

### Outcome measures

The primary outcome in this study was all-cause mortality at 28 days, and the secondary outcome was the all-cause mortality rate during the ICU admission.

### Statistical analysis

The thresholds for SHR and GV were determined a priori based on tertiles of their distributions in the derivation cohort, following established practice in previous critical care studies to account for potential nonlinear associations[18]. Participants were stratified into high vs low groups based on tertiles of SHR and GV (SHR: <0.94, 0.94–1.23, >1.23; GV: <17.63, 17.63–28.56, >28.56), with the highest tertile defined as “high” and the lower two tertiles as “low.” Normality of continuous variables was assessed using the Shapiro–Wilk tests. Normally distributed variables (reported as mean ± SD) were compared via Student’s t-tests or one-way ANOVA, while nonnormally distributed variables (expressed as median and IQR) were analyzed using Wilcoxon rank-sum tests. Categorical variables (presented as counts and percentages) were evaluated by χ^2^ or Fisher’s exact tests. Cumulative all-cause mortality risk was estimated using Kaplan–Meier (KM) curves. Cox proportional hazards regression models were employed to examine associations between SHR, GV, their combined profiles, and mortality: Model 1 (unadjusted), Model 2 (adjusted for age, sex, BMI), and Model 3 (adjusted for multiple factors). The SHR and GV were analyzed with restricted cubic splines (RCS) for dose–effect relationships. Proportional hazards assumptions were verified via Schoenfeld residuals, and landmark analyses were conducted to assess temporal variations in risk profiles.

To further ensure the robustness of our findings and address potential misclassification bias from using a single HbA1c measurement for metabolic grouping, we performed a sensitivity analysis. In this analysis, diabetes status was redefined based on documented medical history and the use of antidiabetic medications during the index hospitalization. Patients meeting either criterion were classified as having DM. Finally, subgroup analyses stratified by sex, age, and comorbidities were conducted, and forest plots were generated to visualize hazard ratios with 95% confidence intervals across subgroups. All analyses were conducted in Python (v3.9.12) and SPSS (v26.0), with two-tailed *P* < 0.05 considered statistically significant.

## Result

### Baseline characteristics

A total of 4838 patients meeting the analytical criteria were identified. The baseline characteristics of the study population are presented in Table 1. Overall, the median age was 69 years, with 3028 patients (62.58%) being female. Of these patients, 3902 (80.7%) survived 28 days following ICU admission, while 936 (19.3%) were nonsurvivors. Compared to survivors, nonsurvivors were older and exhibited a higher prevalence of comorbidities, including AKI, heart failure, hypertension, and COPD. Notably, nonsurvivors were less likely to receive glucose-lowering medications or insulin therapy but had higher mechanical ventilation use. In deceased patients, lower levels of albumin and pH were observed, whereas elevated levels were identified in ALT, AST, BUN, glucose, platelet count, lactate, WBC, creatinine, SHR, and GV.

**Table 1:** Baseline characteristics according to 28-day mortality

Variable	Overall	Survivors (*n* = 3902)	Nonsurvivors (*n* = 936)	*P*-value
Demographics	**–**	**–**	**–**	**–**
Age (years)	68 (58–77)	68 (58–77)	71 (61–81)	<0.01
Male, *n* (%)	3028 (62.59)	2493 (63.89)	535 (57.16)	<0.01
BMI (kg/m^2^)	28.57 (24.78–33.43)	28.77 (24.98–33.55)	27.53 (23.93–32.62)	<0.01
Vital signs	**–**	**–**	**–**	**–**
Abpd (mmHg)	60 (52–69)	61 (53–69)	59 (51–69)	0.02
Abps (mmHg)	117 (103.25–135)	117 (103–133)	119.5 (104–141)	<0.01
Temperature (℃)	36.78 (36.5–37.11)	36.78 (36.5–37.11)	36.78 (36.44–37.11)	0.07
HR (bpm)	85 (75–99)	84 (75–98)	89 (75–104)	<0.01
Comorbidities	**–**	**–**	**–**	**–**
AKI, *n* (%)	2010 (41.55)	1455 (37.29)	555 (59.29)	<0.01
HF, *n* (%)	1649 (34.08)	1263 (32.37)	386 (41.24)	<0.01
HLD, *n* (%)	2292 (47.37)	1927 (49.38)	365 (39.00)	<0.01
HTN, *n* (%)	2196 (45.39)	1865 (47.80)	331 (35.36)	<0.01
COPD, *n* (%)	635 (13.13)	483 (12.38)	152 (16.24)	<0.01
NGR, *n* (%)	1535 (31.73)	1242 (31.83)	293 (31.30)	0.42
Pr-DM, *n* (%)	1165 (24.08)	952 (24.40)	213 (22.76)	**–**
DM, *n* (%)	2138 (44.19)	1708 (43.77)	430 (45.94)	**–**
Treatment	**–**	**–**	**–**	**–**
Hypoglycemic drugs, *n* (%)	514 (10.62)	495 (12.69)	19 (2.03)	<0.01
Mechanical ventilation, *n* (%)	2667 (55.13)	2095 (53.69)	572 (61.11)	<0.01
RI, *n* (%)	2252 (46.55)	1879 (48.15)	373 (39.85)	<0.01
Laboratory measurements				
Albumin (g/dL)	3.2 (2.7–3.6)	3.2 (2.8–3.6)	3 (2.6–3.5)	<0.01
ALT (IU/L)	26 (16–49)	26 (16–48)	27 (15–53)	<0.01
AST (IU/L)	38 (24–77)	37 (24–73)	43 (25–91.25)	<0.01
BUN (mg/dL)	19 (14–30)	18 (13–27)	25 (17–42)	<0.01
Glucose (mg/dL)	131 (109–177)	129 (108–170)	147 (115.75–209.25)	<0.01
HbA1c (%)	5.9 (5.5–6.8)	5.9 (5.5–6.8)	5.9 (5.5–6.7)	0.23
Hb (g/dL)	10.7 (9–12.4)	10.7 (9.1–12.4)	10.7 (8.9–12.5)	0.72
PH	7.39 (7.33–7.43)	7.39 (7.33–7.44)	7.37 (7.3–7.43)	<0.01
Plt (10^9^/L)	180 (131–243)	176.5 (130–239)	194 (133–261.25)	0.01
RBC (m/Ul)	3.58 (3.02–4.19)	3.57 (3.03–4.17)	3.6 (2.96–4.25)	0.42
Lactate (mmol/L)	1.8 (1.2–2.6)	1.8 (1.2–2.5)	1.9 (1.3–3.1)	<0.01
GV (%)	22.26 (15.49–32.49)	21.52 (15.03–31.66)	24.91 (17.56–35.67)	<0.01
SHR	1.07 (0.88–1.34)	1.05 (0.86–1.29)	1.19 (0.94–1.55)	<0.01
WBC (K/UL)	11.9 (8.7–16.1)	11.7 (8.7–15.7)	12.6 (9–17.92)	<0.01
Creatinine (mg/dL)	1 (0.8–1.5)	1 (0.8–1.4)	1.2 (0.9–2)	<0.01
Clinical scores	**–**	**–**	**–**	**–**
SOFA score	5 (3–8)	5 (3–7)	6 (4–9)	<0.01
Charlson score	5 (3–7)	5 (3–7)	7 (5–8)	<0.01

Abpd, arterial blood pressure – diastolic; Abps, arterial blood pressure – systolic; AKI, acute kidney injury; ALT, alanine aminotransferase; AST, aspartate aminotransferase; BMI, body mass index; BUN, blood urea nitrogen; COPD, chronic obstructive pulmonary disease; DM, diabetes mellitus; GCS, Glasgow Coma Scale; GV, glycemic variability; Hb, haemoglobin concentration; HbA1c, glycated hemoglobin; HF, heart failure; HLD, hyperlipidemia; HR, heart rate; HTN, hypertension; NGR, normal glucose regulation; Plt, platelet; Pre-DM, prediabetes mellitus; RBC, red blood cell; SOFA, Sequential Organ Failure Assessment; SHR, stress hyperglycemia ratio; WBC, white blood cell.

### The association between SHR and mortality

**Figure 2.**
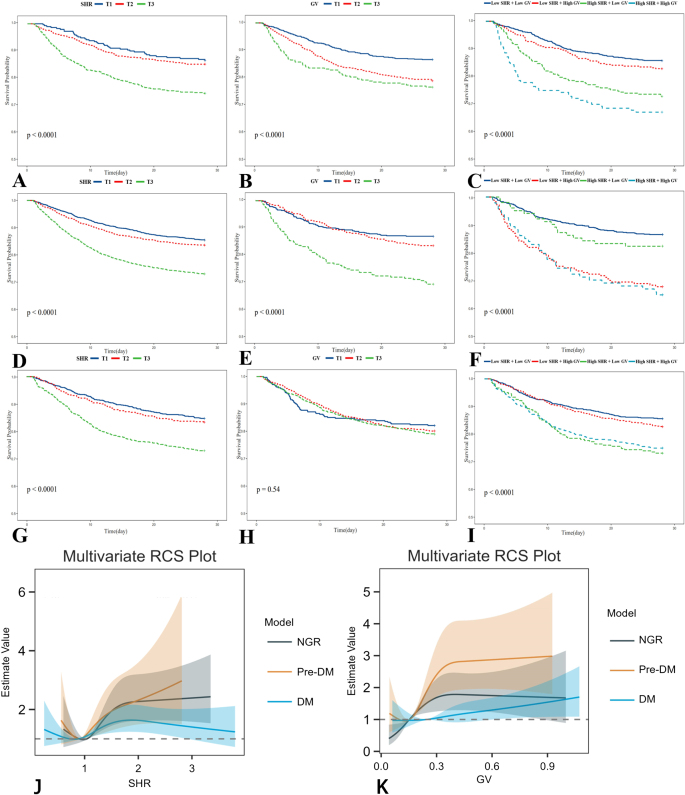
Kaplan–Meier curves of SHR, GV, and their combination for 28-day mortality. (**A–C**) patients with NGR; (**D–F**) patients with Pre-DM; (**G–I**) patients with DM; (**J**) SHR in different populations with glucose metabolic states. NGR: *P* for nonlinear = 0.001, *P* for overall < 0.001; Pre-DM: P for nonlinear = 0.107, *P* for overall < 0.001; DM: *P* for nonlinear = 0.014, *P* for overall < 0.001. (**K**) GV in different populations with glucose metabolic status. NGR: *P* for nonlinear = 0.248, *P* for overall < 0.001; Pre-DM: *P* for nonlinear = 0.029, *P* for overall < 0.001; DM: *P* for nonlinear = 0.908, *P* for overall < 0.001.

SHR, stress hyperglycemia ratio; GV, glycemic variability; NGR, normal glucose regulation; Pre-DM, prediabetes mellitus; DM, diabetes mellitus.

**Figure 3.**
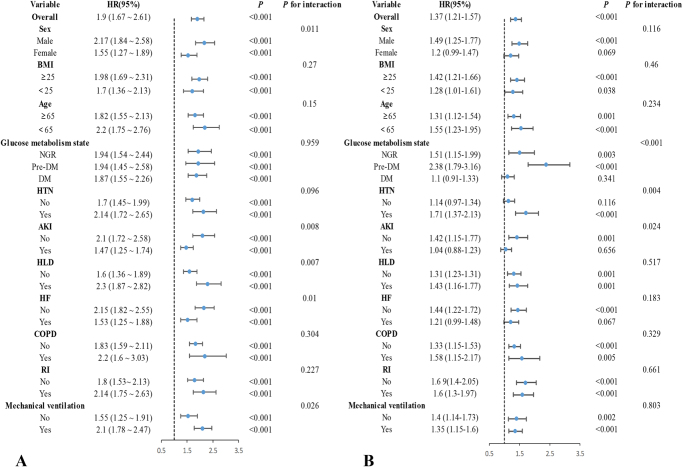
Forest plots for subgroup analyses of (**A**) SHR and (**B**) GV with 28-day mortality.

NGR, normal glucose regulation; Pre-DM, prediabetes mellitus; DM, diabetes mellitus; HTN, hypertension; AKI, acute kidney injury; HLD, hyperlipidemia; HF, heart failure; COPD, chronic obstructive pulmonary disease; RI, Insulin.

During the hospitalization period, 936 patients (19.3%) died, while 641 patients (13.2%) succumbed during their ICU stay. KM curves demonstrated that the 28-day survival rate progressively declined with increasing SHR quantiles across different glucose metabolic states (Fig. 2A, D, and G). In adjusted Cox proportional hazards regression analyses of the overall cohort, patients in the highest SHR tertile exhibited 1.52-fold (HR = 1.52, 95% CI: 1.293–1.798) and 1.54-fold (HR = 1.54, 95% CI: 1.261–1.877) increased risks for 28-day mortality and ICU mortality, respectively, compared to those in the lowest tertile. Similar patterns emerged across distinct glucose metabolism subgroups (Supplementary Digital Content Table S1, available at: http://links.lww.com/JS9/F192). RCS analysis revealed a linear association between SHR and 28-day mortality in Pre-DM individuals (*P* for nonlinearity = 0.107), whereas U-shaped relationships were identified in both NGR and DM populations (Fig. 2J). Subgroup analyses demonstrated significant interactions between SHR and sex, AKI, HTN, HF, and mechanical ventilation status (Fig. 3A).

### The association between GV and mortality

The KM curves illustrating the association between GV and 28-day mortality are presented in Figure 2B, E, and H. Notably, no significant survival differences were observed across GV tertiles in the DM population (*P* = 0.54). Fully adjusted Cox proportional hazards regression analyses demonstrated GV as an independent predictor of in-ICU mortality within the NGR subgroup (*P* < 0.05). Conversely, no significant associations between GV and 28-day or in-ICU mortality emerged in either the overall population (28-day mortality: highest vs lowest tertile HR = 1.195, 95% CI: 0.988–1.446; in-ICU mortality: HR = 1.258, 95% CI: 0.988–1.602) or the DM subgroup (28-day mortality: HR = 0.984, 95% CI: 0.714–1.356; in-ICU mortality: HR = 0.997, 95% CI: 0.638–1.559) (Supplementary Digital Content Table S1, available at: http://links.lww.com/JS9/F192). RCS analysis identified a nonlinear relationship between GV and 28-day mortality in Pre-DM patients (*P* for nonlinearity = 0.029), whereas no distinct dose-response pattern was detected in DM individuals (Fig. 2K). Significant interaction effects were observed between GV and glucose metabolic status, HTN, or AKI regarding 28-day mortality (*P* for interaction < 0.05) (Fig. 3B).

### The association of the combination of SHR and GV with mortality

The KM curves depicting 28-day mortality for the combination of SHR and GV are shown in Figure 2C, F, and I. The subgroup with combined high SHR and high GV exhibited markedly lower survival compared to other combinations, but similar survival rates were observed between high SHR/high GV and low SHR/high GV subgroups in the Pre-DM population. Among NGR patients, those with elevated SHR (>1.23) and GV (>28.56) demonstrated the highest risks for 28-day mortality (HR = 2.06, 95% CI: 1.40–3.04) and ICU mortality (HR = 2.78, 95% CI: 1.73–4.46). Similarly, Pre-DM individuals with high SHR/high GV showed the greatest ICU mortality risk (HR = 1.94, 95% CI: 1.15–3.27), while the low SHR/high GV subgroup (SHR < 1.23, GV > 28.56) exhibited the highest 28-day mortality risk (HR = 2.45, 95% CI: 1.73–3.48). In DM patients, the high SHR/high GV combination was associated with peak ICU mortality (HR = 1.59, 95% CI: 1.12–2.26), whereas high SHR/low GV (SHR > 1.23, GV < 28.56) conferred the greatest 28-day mortality risk (HR = 1.46, 95% CI: 1.06–2.01) (Table 2). RCS analysis in the total population revealed a U-shaped relationship between SHR and the overall population, whereas GV showed a linear association (*P* for nonlinearity = 0.186, Supplementary Digital Content Figure S1, available at: http://links.lww.com/JS9/F192).

**Table 2:** The association of the combination of SHR and GV with 28-day mortality

Variables	Model 1		Model 2		Model 3	
	HR (95%CI)	*P*	HR (95%CI)	*P*	HR (95%CI)	*P*
28-day mortality						
Overall						
Group 1	1.00 (Reference)		1.00 (Reference)		1.00 (Reference)	
Group 2	1.45 (1.23–1.71)	<0.001	1.45 (1.23–1.71)	<0.001	1.26 (1.06–1.50)	0.01
Group 3	1.97 (1.63–2.39)	<0.001	1.99 (1.64–2.41)	<0.001	1.48 (1.22–1.80)	<0.001
Group 4	2.24 (1.89–2.67)	<0.001	2.26 (1.90–2.69)	<0.001	1.60 (1.32–1.95)	<0.001
*P* for trend	1.32 (1.25–1.39)	<0.001	1.32 (1.25–1.40)	<0.001	1.17 (1.11–1.25)	<0.001
Patients with NGR						
Group 1	1.00 (Reference)		1.00 (Reference)		1.00 (Reference)	
Group 2	1.22 (0.89–1.68)	0.214	1.20 (0.88–1.65)	0.252	1.00 (0.71–1.37)	0.929
Group 3	2.09 (1.56–2.80)	<0.001	2.11 (1.57–2.84)	<0.001	1.67 (1.22–2.29)	0.001
Group 4	2.75 (1.96–3.87)	<0.001	2.88 (2.03–4.08)	<0.001	2.06 (1.40–3.04)	<0.001
*P* for trend	1.42 (1.28–1.57)	<0.001	1.44 (1.29–1.60)	<0.001	1.28 (1.14–1.44)	<0.001
Patients with Pre-DM						
Group 1	1.00 (Reference)		1.00 (Reference)			
Group 2	2.74 (1.98–3.78)	<0.001	2.65 (1.92–3.66)	<0.001	2.45 (1.73–3.48)	<0.001
Group 3	1.34 (0.81–2.20)	<0.001	1.37 (0.83–2.26)	<0.001	1.01 (0.60–1.72)	0.97
Group 4	2.98 (2.01–4.40)	<0.001	2.97 (2.01–4.40)	<0.001	2.26 (1.44–3.53)	<0.001
*P* for trend	1.40 (1.24–1.57)	<0.001	1.40 (1.24–1.57)	<0.001	1.26 (1.10–1.44)	0.001
Patients with DM						
Group 1	1.00 (Reference)		1.00 (Reference)		1.00 (Reference)	
Group 2	1.22 (0.94–1.57)	0.135	1.23 (0.95–1.58)	0.117	1.10 (0.84–1.45)	0.498
Group 3	2.03 (1.49–2.76)	<0.001	1.96 (1.44–2.67)	<0.001	1.46 (1.06–2.01)	0.02
Group 4	1.89 (1.46–2.43)	<0.001	1.85 (1.43–2.39)	<0.001	1.36 (1.02–1.80)	0.034
*P* for trend	1.26 (1.16–1.36)	<0.001	1.24 (1.15–1.35)	<0.001	1.12 (1.03–1.22)	0.011
In-ICU mortality						
Overall						
Group 1	1.00 (Reference)	**–**	1.00 (Reference)		1.00 (Reference)	**–**
Group 2	0.98 (0.80–1.21)	0.872	0.98 (0.80–1.21)	0.854	1.05 (0.84–1.30)	0.684
Group 3	1.31 (1.02–1.66)	0.021	1.33 (1.05–1.67)	0.018	1.30 (1.02–1.66)	0.033
Group 4	1.83 (1.48–2.25)	<0.001	1.85 (1.50–2.27)	<0.001	1.92 (1.53–2.42)	<0.001
*P* for trend	1.32 (1.25–1.39)	<0.001	1.24 (1.15–1.32)	<0.001	1.24 (1.15–1.34)	<0.001
Patients with NGR						
Group 1	1.00 (Reference)		1.00 (Reference)		1.00 (Reference)	**–**
Group 2	1.04 (0.70–1.55)	0.847	1.04 (1.70–1.54)	0.858	1.12 (0.74–1.68)	0.592
Group 3	1.57 (1.08–2.29)	0.018	1.57 (1.06–2.29)	0.02	1.61 (1.07–2.46)	0.023
Group 4	2.91 (1.96–4.33)	<0.001	2.91 (1.95–4.37)	<0.001	2.78 (1.73–4.46)	<0.001
*P* for trend	1.40 (1.23–1.60)	<0.001	1.438 (1.29–1.60)	<0.001	1.28 (1.13–1.44)	<0.001
Patients with Pre-DM						
Group 1	1.00 (Reference)		1.00 (Reference)		1.00 (Reference)	
Group 2	1.83 (1.25–2.68)	0.002	1.74 (1.18–2.56)	0.005	1.75 (1.13–2.70)	0.012
Group 3	0.87 (0.49–1.55)	0.632	0.88 (0.49–1.57)	0.663	0.90 (0.47–1.68)	0.718
Group 4	1.88 (1.20–2.95)	0.006	1.93 (1.26–3.03)	0.004	1.94 (1.15–3.27)	0.013
*P* for trend	1.17 (1.02–1.35)	0.029	1.18 (1.02–1.36)	0.023	1.19 (1.01–1.40)	0.04
Patients with DM						
Group 1	1.00 (Reference)	**–**	1.00 (Reference)	**–**	1.00 (Reference)	**–**
Group 2	0.72 (0.52–0.99)	0.048	0.73 (0.53–1.02)	0.064	0.80 (0.55–1.15)	0.23
Group 3	1.32 (0.92–1.90)	0.135	1.36 (0.94–1.95)	0.103	1.41 (0.96–2.08)	0.082
Group 4	0.44 (1.06–1.96)	0.021	1.45 (1.06–1.97)	0.019	1.59 (1.12–2.26)	0.01
*P* for trend	1.20 (1.09–1.33)	<0.001	1.20 (1.09–1.33)	<0.001	1.25 (1.12–1.39)	<0.001

Model 1: unadjusted;

Model 2: adjusted for age, sex and BMI;

Model 3: adjusted for Model 2 plus SOFA score, GCS score, Charlson score, HR, Abps, Abpd, Albumin, Lactate, PH, ALT, AST, Creatinine, BUN, Hemoglobin, Plt, RBC, WBC, HTN, AKI, HLD, HF, COPD, RI, Hypoglycemic drugs, Mechanical ventilation, Temperature.

Group 1: Low SHR and Low GV (SHR < 1.23 and GV < 28.56); Group 2: Low SHR and High GV (SHR < 1.23 and GV > 28.56); Group 3: High SHR and Low GV (SHR > 1.23 and GV < 28.56); Group 4: High SHR and High GV (SHR > 1.23 and GV > 28.56).

The power analysis showed that the high-risk combinations of low SHR + high GV and high SHR + high GV were adequately powered (>0.99 for 28-day and >0.95 for ICU mortality), suggesting that the observed associations are unlikely to be due to chance. In contrast, the high SHR + low GV combination exhibited limited statistical power, which is consistent with its smaller effect size and confidence intervals crossing unity (Supplementary Digital Content Figure S2, available at: http://links.lww.com/JS9/F192).

In fully adjusted Cox models, the SHR × GV interaction showed heterogeneous effects across glycemic groups. In NGR patients, it was associated with a higher 28-day mortality risk (HR = 1.29, 95% CI 1.02–1.64), whereas in Pre-DM patients, it was inversely associated with both 28-day and ICU mortality, indicating a possible risk-attenuating effect. In DM patients, the interaction was small and nonsignificant (Supplementary Digital Content Table S2, available at: http://links.lww.com/JS9/F192). These findings suggest that the joint influence of stress hyperglycemia and glycemic variability on outcomes is modified by baseline glycemic status.

**Figure 4.**
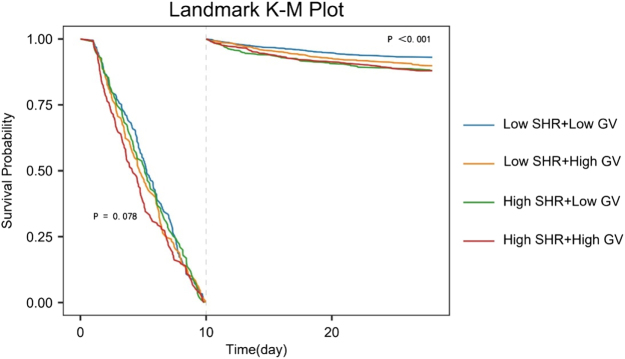
Survival landmark analyses of (**A**) SHR and (**B**) GV with 28-day mortality. SHR, stress hyperglycemia ratio; GV, glycemic variability.

Proportional hazards testing revealed nonconstant risk ratios between SHR/GV combinations and mortality over time (*P* = 0.025, Supplementary Digital Content Table S3, available at: http://links.lww.com/JS9/F192). The confidence interval for the Beta value approached zero around day 10 (Supplementary Digital Content Figure S3, available at: http://links.lww.com/JS9/F192). As shown in Figure 4, no significant survival differences were observed among subgroups within 10 days post-ICU admission (*P* = 0.078). However, beyond 10 days, survival disparities became pronounced (*P* < 0.001), with the low SHR/low GV subgroup demonstrating superior survival compared to other combinations.

### Sensitivity analysis

In the sensitivity analysis using diabetes status redefined by medical history and medication records, the associations of SHR, GV, and their combinations with 28-day and ICU mortality remained consistent with the primary results (Supplementary Digital Content Table S4, available at: http://links.lww.com/JS9/F192). These findings indicate that our conclusions are robust to alternative definitions of diabetes status.

### Machine learning: SHR, GV, and 28-day mortality

**Figure 5.**
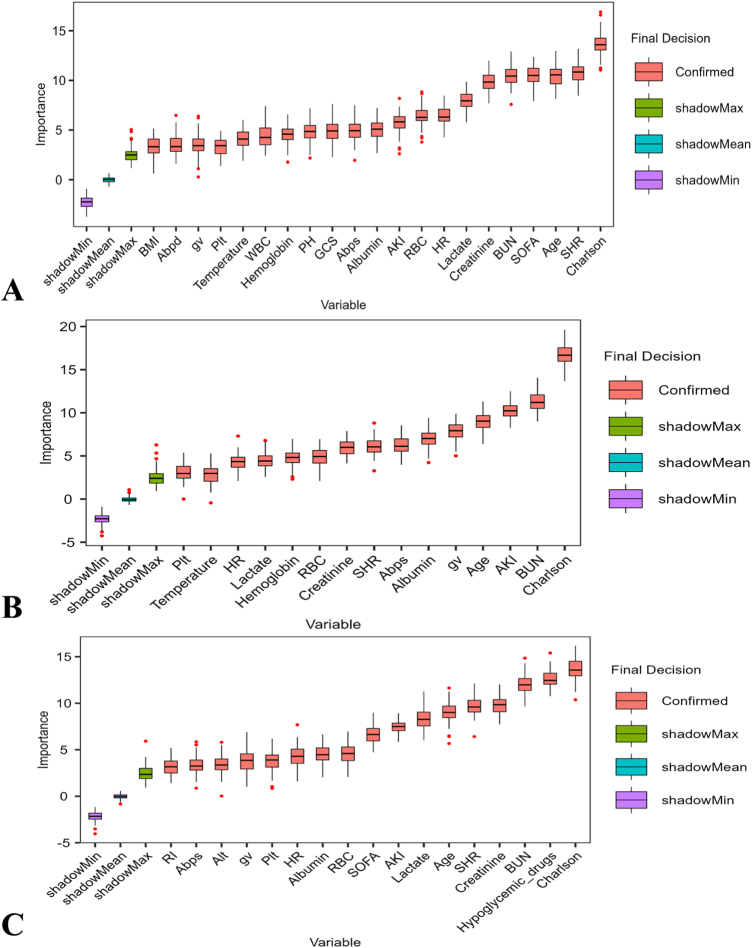
The Boruta algorithm ranks the importance of potential risk factors for 28-day mortality. The horizontal axis represents the name of each variable, and the vertical axis represents the *Z*-value of each variable. The box plot shows the *Z*-values of each variable during the model training period. (**A**) NGR patients, (**B**) Pre-DM patients, and (**C**) DM patients.

BMI, body mass index; Abpd, arterial blood pressure (diastolic); GV, glycemic variability; Plt, platelet count; WBC, white blood cell count; PH, potential of hydrogen; GCS, Glasgow coma scale; Abps, arterial blood pressure (systolic); AKI, acute kidney injury; RBC, red blood cell count; HR, heart rate; BUN, blood urea nitrogen; SOFA, sequential organ failure assessment; SHR, stress hyperglycemia ratio; Albumin, serum albumin; Lactate, lactate; Creatinine, creatinine; Charlson, Charlson comorbidity index; NGR, normal glucose regulation; Pre-DM, prediabetes mellitus; DM, diabetes mellitus.

The final variables incorporated into the machine learning models were identified through Boruta algorithm analysis (Fig. 5), with variable importance displayed in descending order from right to left. The Boruta algorithm selected 21, 15, and 17 features as optimal predictors of mortality from the NGR, Pre-DM, and DM subgroups, respectively. The results of the Boruta algorithm in the total population are shown in Supplementary Digital Content Figure S4, available at: http://links.lww.com/JS9/F192.

**Figure 6.**
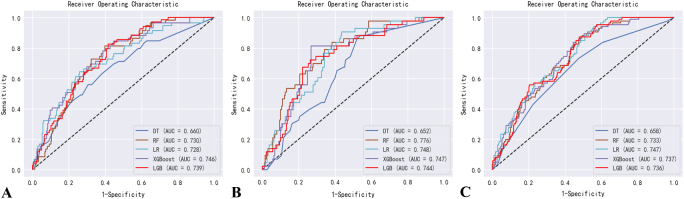
Receiver operating characteristic curve of five ML models for predicting 28-day mortality. (A) NGR patients, (B) Pre-DM patients, and (C) DM patients.

DT, decision tree; RF, random forest; LR, logistic regression; XGBoost, extreme gradient boosting; LGB, light gradient boosting machine; AUC, area under the receiver operating characteristic curve.

The XGBoost model demonstrated superior performance in the NGR population (AUC = 0.746; sensitivity: 0.847, specificity: 0.552, accuracy: 0.808, F1-score: 0.552) (Fig. 6A). The RF model exhibited the best predictive capability in the Pre-DM subgroup (AUC = 0.747; sensitivity: 0.942, specificity: 0.442, accuracy: 0.617, F1-score: 0.556) (Fig. 6B). For the DM cohort, the LR model achieved optimal performance (AUC = 0.751; sensitivity: 0.834, specificity: 0.557, accuracy: 0.808, F1-score: 0.486) (Fig. 6C). Detailed sensitivity, specificity, and accuracy metrics for other models are provided in Table 3. The results of the ROC curve analysis in the total population are presented in Supplementary Digital Content Figure S5, available at: http://links.lww.com/JS9/F192. In the Pre-DM group, the Random Forest model showed a higher AUC compared with SOFA (0.776 vs 0.540), with an IDI of 0.042.

**Table 3:** The performance comparison of each ML model in predicting 28-day mortality

	AUC	Sensitivity	Specificity	Accuracy	F1
Overall	–	–	–	–	–
LR	0.727	0.759	0.621	0.805	0.539
DT	0.555	0.283	0.826	0.721	0.554
RF	0.518	0.043	0.994	0.81	0.487
XGBoost	0.751	0.834	0.557	0.808	0.486
LGB	0.717	0.036	0.990	0.799	0.477
NGR	–	–	–	–	–
LR	0.728	0.644	0.742	0.684	0.612
DT	0.66	0.559	0.694	0.801	0.557
RF	0.73	0.814	0.601	0.798	0.444
XGBoost	0.746	0.847	0.552	0.808	0.552
LGB	0.739	0.043	0.992	0.779	0.478
Pre-DM	–	–	–	–	–
LR	0.748	0.907	0.563	0.665	0.584
DT	0.652	0.884	0.458	0.768	0.483
RF	0.776	0.837	0.616	0.811	0.448
XGBoost	0.747	0.814	0.732	0.781	0.49
LGB	0.744	0.721	0.737	0.79	0.538
DM	–	–	–	–	–
LR	0.747	0.942	0.442	0.617	0.566
DT	0.658	0.733	0.523	0.799	0.444
RF	0.733	0.884	0.506	0.799	0.444
XGBoost	0.737	0.837	0.556	0.799	0.476
LGB	0.736	0.872	0.52	0.787	0.506

**Figure 7.**
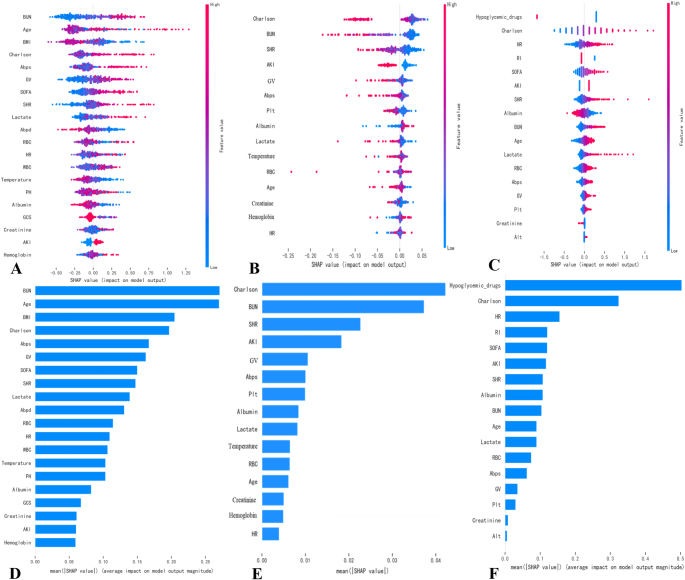
Interpretation of a 28-day mortality prediction model using ML. (A and D) NGR patients, (B and E) Pre-DM patients, and (C and F) DM patients.

BMI, body mass index; Abpd, arterial blood pressure diastolic; GV, glycemic variability; Plt, platelet count; WBC, white blood cell count; PH, potential of hydrogen (blood pH); GCS, Glasgow Coma Scale; Abps, arterial blood pressure systolic; Albumin, serum albumin; AKI, acute kidney injury; RBC, red blood cell count; HR, heart rate; Lactate, serum lactate; Creatinine, serum creatinine; BUN, blood urea nitrogen; SOFA, Sequential Organ Failure Assessment; Age, patient age; SHR, stress hyperglycemia ratio; Charlson, Charlson Comorbidity Index.

Figure 7A and D presents the scatter plots of mortality-associated risk factors and mean importance bar plots identified by the XGBoost model in the NGR subgroup. Figure 7B and E demonstrates the scatter plots of mortality-associated risk factors and corresponding mean importance bar plots determined by the RF model within the Pre-DM subgroup. Similarly, Figure 7C and F displays the scatter plots of mortality-associated risk factors and associated mean importance bar plots ascertained by the RF model in the DM population. The interpretation of the 28-day mortality prediction model in the total patient population is presented in Supplementary Digital Content Figure S6, available at: http://links.lww.com/JS9/F192. To validate the models’ interpretability, Supplementary Digital Content Figure S7, available at: http://links.lww.com/JS9/F192 provides a comparative visualization of clinical parameters influencing mortality risk predictions between representative survivors (Supplementary Digital Content Figure S7A, C, E, available at: http://links.lww.com/JS9/F192) and nonsurvivors (Supplementary Digital Content Figure S7B, D, F, available at: http://links.lww.com/JS9/F192).

## Discussion

In this study, SHR consistently predicted mortality across all glucose metabolic states, whereas GV showed weaker associations in DM patients. Patients with high SHR and GV experienced the greatest ICU mortality risk, with distinct 28-day mortality patterns observed in NGR, Pre-DM, and DM subgroups. Landmark analysis indicated that the prognostic separation of SHR and GV emerged after approximately 10 ICU days, suggesting a time-dependent effect. Machine learning-based feature selection and interpretability analysis further validated SHR and GV as robust prognostic determinants of mortality risk. These results support their integration into risk stratification frameworks for sepsis and highlight the potential importance of combining acute and chronic glycemic markers in prognostic modeling.

Current research on SHR and GV has primarily focused on critically ill populations. Studies have established SHR as an independent predictor of mortality, effectively assessing the relationship between acute hyperglycemia and chronic metabolic adaptation, thereby enabling more precise mortality risk stratification in this population[7]. Yuwen Chen *et al* utilized the MIMIC-IV database to assess the prognostic value of SHR for all-cause mortality in critically ill patients with cerebrovascular diseases, demonstrating a stronger association in nondiabetic individuals[23]. Cancan Cui *et al* highlighted the potential utility of SHR in diabetes risk stratification and prognosis, outperforming conventional metrics such as glucose and HbA1c[24]. Evidence suggests that long-term glycemic variability in fasting glucose serves as an independent predictor of mortality in patients with type 2 diabetes[25]. Hao-ming He *et al* evaluated the combined prognostic significance of SHR and GV in coronary heart disease patients, revealing that nondiabetic individuals with elevated SHR and GV exhibited the poorest outcomes, whereas diabetic patients with high SHR but low GV demonstrated the highest mortality risk. In septic patients, the mortality impact of hyperglycemia and elevated GV intensified with sepsis severity[26]. These findings collectively suggest that integrated assessment of GV and SHR may enhance clinical outcome prediction in critically ill populations and inform tailored glycemic management strategies across heterogeneous glucose metabolic phenotypes.

Recent studies have demonstrated the feasibility of integrating ML algorithms into ICU workflows using readily available physiological metrics. For example, Rostamzadeh *et al* applied K-nearest neighbors to handgrip strength and endurance time, accurately predicting COVID-19 mortality among older ICU patients with minimal computational and resource requirements[27]. Similarly, in a large-scale ICU cohort, the same group compared Alpha and Delta COVID-19 variants and confirmed the prognostic value of handgrip strength using ML-based analysis, reinforcing the clinical utility of low-cost, bedside measurements[28]. These findings suggest that ML models based on easily obtainable parameters – such as our SHR and GV – can be seamlessly embedded into ICU electronic health record systems to provide continuous, automated risk stratification. Such tools could help clinicians identify high-risk patients earlier, guide targeted glucose management strategies, and optimize resource allocation, thereby improving patient outcomes without adding substantial operational burden.

Evidence suggests that circulating levels of proinflammatory cytokines are modulated by glucose, and heightened oxidative stress may link stress hyperglycemia to cardiovascular events via enhanced cytokine production[29]. In diabetes, impaired nitric oxide (NO)-mediated antiaggregatory responses contribute to platelet dysfunction, partially explaining hypercoagulability in hyperglycemic states[30]. Glucose-containing parenteral nutrition in infected infants is associated with prolonged hospitalization, potentially by altering immune-metabolic crosstalk or hepatic metabolism to favor glycolysis over active immune defense[31]. Patients with ≥5 years of T2DM exhibit elevated sepsis risk, likely attributable to chronic inflammation from intermittent/persistent hyperglycemia exacerbating immune dysregulation[32]. In T2DM, advanced glycation end-products (AGEs) interact with RAGE via MyD88 and TLR4, activating NF-κB – a driver of hyperinflammation, cytokine storms, and organ failure in sepsis^[1,33]^. Insulin resistance in T2DM upregulates immune-related genes, while obesity-associated tissue inflammation progressively heightens sepsis susceptibility[34].

This study observed consistent mortality associations with elevated SHR across all glucose metabolic states, aligning with findings by Fengjuan Yan *et al*[35]. Stress hyperglycemia may induce endothelial mitochondrial ROS overproduction, precipitating dysfunction[36]. Acute glucose spikes promote endothelial injury and aberrant coagulation activation, potentially exacerbating sepsis-related DIC[37]. Both stress hyperglycemia and hypoglycemia correlate with sepsis mortality, with high GV patients potentially exposed to dual insults[38]. GV’s association with urinary 8-iso-PGF2α implicates oxidative stress in sepsis progression, possibly mediated by endothelial dysfunction-linked end-organ damage^[39,40]^. Similar to the results of previous studies, this study found that there was no significant correlation between GV and mortality in patients with DM^[4,41–43]^. This may reflect the adaptive tolerance of DM patients to blood glucose fluctuations[44]. Diabetics exhibit higher glycemic thresholds for harm (lower for hypoglycemia, higher for hyperglycemia), enabling broader glucose tolerance than nondiabetics[18]. Notably, elevated GV predicts adverse outcomes only in nondiabetics, while paradoxically trending toward protective effects in diabetics^[18,45]^. In diabetic patients, chronic adaptation to long-standing glycemic variability may blunt the prognostic impact of GV alone, whereas a sudden acute rise in SHR on a relatively stable background (i.e., high SHR combined with low GV) may reflect a stress-related disturbance and therefore confer disproportionately higher short-term mortality risk. Our landmark analysis indicated that the prognostic divergence of SHR and GV becomes more evident after approximately 10 days of ICU stay. This finding suggests that the predictive value of these glycemic parameters is not temporally uniform, but rather time-dependent. During the early phase of ICU admission, SHR and GV alone may provide limited prognostic discrimination, likely due to the influence of acute physiological fluctuations, stress responses, and ongoing therapeutic interventions[46]. Therefore, early risk stratification should consider integrating SHR and GV with additional clinical indicators such as severity scores, organ function markers, and comorbidities to enhance predictive accuracy. In contrast, in the later ICU course, persistent abnormalities in SHR and GV may reflect sustained metabolic dysregulation and underlying pathophysiological instability, thereby offering stronger prognostic signals[18]. This temporal pattern underscores the need for dynamic risk modeling approaches that incorporate time-varying covariates, rather than relying solely on static measurements, to better inform personalized ICU glucose management strategies.

In our analysis, we observed that the discriminative performance of ML models did not substantially exceed that of conventional logistic regression in certain subgroups. However, our intent was not solely to achieve incremental gains in AUC, but to explore the broader utility of ML in modeling complex, nonlinear relationships between SHR, GV, and other clinical covariates. By leveraging SHAP-based interpretability, the ML framework revealed subgroup-specific predictors and their relative contributions, offering clinical insights that conventional regression cannot easily provide^[47,48]^. Furthermore, the flexible architecture of ML models allows future incorporation of temporal glucose trends, inflammatory markers, and external datasets, which may yield greater performance improvements in larger and more heterogeneous populations^[49,50]^. From a clinical perspective, the stable sensitivity observed across probability thresholds supports the use of ML models as a screening tool to avoid missing high-risk patients, even at the cost of some reduction in specificity[51]. This aligns with the prioritization of patient safety in critical care settings. Future research focusing on external validation and multimodal data integration is proposed to assess and enhance the added value of machine learning for ICU prognostication.

### Strengths and limitations

Compared with prior studies based on the MIMIC-IV database, our work offers three key innovations. First, we are the first to phenotype-stratify sepsis patients by glucose metabolic status and reveal that the highest-risk SHR/GV combinations differ substantially across phenotypes. Second, we introduce landmark analysis to demonstrate that the prognostic impact of combined SHR and GV emerges significantly after ICU day 10, suggesting a novel temporal dimension to glycemic dysregulation in sepsis. Third, by integrating Boruta-based feature selection with SHAP interpretability, we achieve both strong predictive performance and clinically relevant transparency, enabling the identification of subgroup-specific predictor hierarchies. These methodological and clinical advances fill an important gap in dynamic glycemic risk assessment for sepsis and provide a feasible pathway toward personalized glycemic management in critical care.

This investigation has several limitations. First, as this study was observational, no causal relationship between glycemic parameters and outcomes can be inferred. While SHR and GV showed prognostic associations, glucose management strategies based on them remain hypothetical and require confirmation in prospective trials. Our findings are hypothesis-generating and intended to guide future research, not direct clinical practice. Second, selection bias may exist due to the exclusion of patients lacking HbA1c data or insufficient glycemic measurements. Third, as with all observational studies, residual confounding cannot be fully excluded despite extensive multivariable adjustment. Finally, this study lacked external validation in independent datasets, which may limit the generalizability of our findings to other ICU populations. Future studies should aim to replicate these results in diverse cohorts and settings to strengthen their applicability.

## Conclusion

This study suggests that the combined assessment of SHR and GV may serve as a useful prognostic tool for mortality risk stratification in sepsis patients, with enhanced predictive accuracy observed in NGR and Pre-DM subgroups. These findings highlight the potential value of personalized glycemic management strategies tailored to individual metabolic phenotypes, which should be explored and validated in future prospective studies.

## Supplementary Material

## Data Availability

Data were obtained from the publicly available MIMIC-IV database (https://mimic.mit.edu/), and the SQL/Python code used in this study is provided as Supplementary Material.

## Research registration unique identifying number (UIN)

This study is a retrospective study, so registration was waived.
