# Supplementary material for: Simultaneous assessment of stress hyperglycemia ratio and glucose variability to predict all-cause mortality in sepsis patients across different glucose metabolic states: an observational cohort study with interpretable machine learning approach
Source: Int J Surg. 2025 Sep 23;112(1):1219–32. doi: 10.1097/JS9.0000000000003525 (PMC12825658; doi:10.1097/JS9.0000000000003525)
Supplement: Supplementary file 1 [file js9-112-1219-001.docx]

Table S1 The association of the SHR and GV with 28-day and ICU mortality

| **Variables** | | **Model 1** | | |  | | **Model 2** | | | |  | | | **Model 3** | | | |
| --- | --- | --- | --- | --- | --- | --- | --- | --- | --- | --- | --- | --- | --- | --- | --- | --- | --- |
|  |  |  |  | |  | |  | |  | |  | | |  | |  | |
|  |  | **HR (95%CI)** | **P** | |  | | **HR (95%CI)** | | **P** | |  | | | **HR (95%CI)** | | **P** | |
| **28-day mortality** | |  |  | |  | |  | |  | |  | | |  | |  | |
| **Overall** | |  |  | |  | |  | |  | |  | | |  | |  | |
| SHR | |  |  | |  | |  | |  | |  | | |  | |  | |
| T1 | | 1.00 (Reference) |  | |  | | 1.00 (Reference) | |  | |  | | | 1.00 (Reference) | |  | |
| T2 | | 1.15 (0.964 ~ 1.371) | 0.12 | |  | | 1.154 (0.968 ~ 1.376) | | 0.11 | |  | | | 1.09 (0.912 ~ 1.303) | | 0.344 | |
| T3 | | 2.038 (1.738 ~ 2.389) | <0.001 | |  | | 2.058 (1.755 ~ 2.414) | | <0.001 | |  | | | 1.524 (1.293 ~ 1.798) | | <0.001 | |
| P for trend | | 1.46 (1.346 ~ 1.584) | <0.001 | |  | | 1.468 (1.353 ~ 1.592) | | <0.001 | |  | | | 1.248 (1.149~ 1.356) | | <0.001 | |
| GV | |  |  | |  | |  | |  | |  | | |  | |  | |
| T1 | | 1.00 (Reference) |  | |  | | 1.00 (Reference) | |  | |  | | | 1.00 (Reference) | |  | |
| T2 | | 1.38 (1.168 ~ 1.631) | <0.001 | |  | | 1.364 (1.154 ~ 1.612) | | <0.001 | |  | | | 1.075 (0.903 ~ 1.28) | | 0.414 | |
| T3 | | 1.634 (1.389 ~ 1.922) | <0.001 | |  | | 1.624 (1.38 ~ 1.912) | | <0.001 | |  | | | 1.195 (0.988 ~ 1.446) | | 0.067 | |
| P for trend | | 1.271 (1.174 ~ 1.376) | <0.001 | |  | | 1.268 (1.171 ~ 1.373) | | <0.001 | |  | | | 1.094 (0.995~ 1.204) | | 0.064 | |
| **Patients with NGR** | |  |  | |  | |  | |  | |  | | |  | |  | |
| SHR | |  |  | |  | |  | |  | |  | | |  | |  | |
| T1 | | 1.00 (Reference) |  | |  | | 1.00 (Reference) | |  | |  | | | 1.00 (Reference) | |  | |
| T2 | | 1.127 (0.778 ~ 1.633) | 0.527 | |  | | 1.185 (0.817 ~ 1.718) | | 0.372 | |  | | | 1.27 (0.864 ~ 1.867) | | 0.224 | |
| T3 | | 2.108 (1.484 ~ 2.995) | <0.001 | |  | | 2.206 (1.549 ~ 3.141) | | <0.001 | |  | | | 1.825 (1.255 ~ 2.653) | | 0.002 | |
| P for trend | | 1.566 (1.322 ~ 1.854) | <0.001 | |  | | 1.587 (1.34 ~ 1.88) | | <0.001 | |  | | | 1.374 ( 1.154~ 1.636) | | <0.001 | |
| GV | |  |  | |  | |  | |  | |  | | |  | |  | |
| T1 | | 1.00 (Reference) |  | |  | | 1.00 (Reference) | |  | |  | | | 1.00 (Reference) | |  | |
| T2 | | 1.652 (1.274 ~ 2.143) | <0.001 | |  | | 1.629 (1.256 ~ 2.114) | | <0.001 | |  | | | 1.145 (0.866 ~ 1.514) | | 0.341 | |
| T3 | | 1.923 (1.409 ~ 2.624) | <0.001 | |  | | 1.889 (1.378 ~ 1.259) | | <0.001 | |  | | | 1.282 (0.906 ~ 1.816) | | 0.161 | |
| P for trend | | 1.411 (1.217 ~ 1.636) | <0.001 | |  | | 1.399 (1.203 ~ 1.626) | | <0.001 | |  | | | 1.134 (0.955 ~ 1.346) | | 0.151 | |
| **Patients with Pre-DM** | | |  | |  | |  | |  | |  | | |  | |  | |
| SHR | |  |  | |  | |  | |  | |  | | |  | |  | |
| T1 | | 1.00 (Reference) |  | |  | | 1.00 (Reference) | |  | |  | | | 1.00 (Reference) | |  | |
| T2 | | 1.37 (0.984 ~ 1.908) | 0.062 | |  | | 1.363 (0.979 ~ 1.897) | | 0.067 | |  | | | 1.356 (0.965 ~ 1.907) | | 0.08 | |
| T3 | | 2.286 (1.624 ~ 3.218) | <0.001 | |  | | 2.339 (1.66 ~ 3.296) | | <0.001 | |  | | | 1.836 (1.264 ~ 2.666) | | 0.001 | |
| P for trend | | 1.516 (1.273 ~ 1.804) | <0.001 | |  | | 1.533 (1.287 ~ 1.827) | | <0.001 | |  | | | 1.348( 1.119~ 1.625) | | 0.002 | |
| GV | |  |  | |  | |  | |  | |  | | |  | |  | |
| T1 | | 1.00 (Reference) |  | |  | | 1.00 (Reference) | |  | |  | | | 1.00 (Reference) | |  | |
| T2 | | 1.262 (0.908 ~ 1.754) | 0.165 | |  | | 1.227 (0.882 ~ 1.707) | | 0.224 | |  | | | 0.753 (0.52 ~ 1.091) | | 0.133 | |
| T3 | | 2.645 (1.908 ~ 3.667) | <0.001 | |  | | 2.528 (1.818 ~ 3.516) | | <0.001 | |  | | | 1.656 (1.127 ~ 2.435) | | 0.01 | |
| P for trend | | 1.624 (1.369 ~ 1.972) | <0.001 | |  | | 1.588 (1.336 ~ 1.887) | | <0.001 | |  | | | 1.305 (1.063 ~ 1.602) | | 0.011 | |
| **Patients with DM** | |  |  | |  | |  | |  | |  | | |  | |  | |
| SHR | |  |  | |  | |  | |  | |  | | |  | |  | |
| T1 | | 1.00 (Reference) |  | |  | | 1.00 (Reference) | |  | |  | | | 1.00 (Reference) | |  | |
| T2 | | 1.106 (0.843 ~ 1.45) | 0.467 | |  | | 1.09 (0.831 ~ 1.43) | | 0.534 | |  | | | 0.941 (0.712 ~ 1.242) | | 0.666 | |
| T3 | | 1.946 (1.565 ~ 2.42) | <0.001 | |  | | 1.885 (1.515 ~ 2.345) | | <0.001 | |  | | | 1.387 (1.104 ~ 1.742) | | 0.005 | |
| P for trend | | 1.413 (1.265 ~ 1.58) | | | <0.001 | |  | | 1.391 (1.244 ~ 1.555) | | <0.001 | |  | 1.192( 1.061~ 1.339) | | 0.003 | |
|  | Table S1 (continued) | | | | |  | |  | | | |  |  | | | |  |
| **Variables** | **Model 1** | | | | |  | | **Model 2** | | | |  | **Model 3** | | | |  |
|  |  | | |  | |  | |  | |  | |  |  | |  | |  |
|  | **HR (95%CI)** | | | **P** | |  | | **HR (95%CI)** | | **P** | |  | **HR (95%CI)** | | **P** | |  |
| GV |  | | |  | |  | |  | |  | |  |  | |  | |  |
| T1 | 1.00 (Reference) | | |  | |  | | 1.00 (Reference) | |  | |  | 1.00 (Reference) | |  | |  |
| T2 | 1.096 (1.809 ~ 1.484) | | | 0.554 | |  | | 1.09 (0.805 ~ 1.477) | | 0.577 | |  | 1.04 (0.759 ~ 1.427) | | 0.806 | |  |
| T3 | 1.167 (0.88 ~ 1.546) | | | 0.283 | |  | | 1.166 (0.879 ~ 1.548) | | 0.286 | |  | 0.984 (0.714 ~ 1.356) | | 0.923 | |  |
| P for trend | 1.076 (0.945 ~ 1.226) | | | 0.268 | |  | | 1.078 (0.945 ~ 1.228) | | 0.264 | |  | 0.982 (0.844 ~ 1.141) | | 0.809 | |  |
| **In-ICU mortality** |  | | |  | |  | |  | |  | |  |  | |  | |  |
| **Overall** |  | | |  | |  | |  | |  | |  |  | |  | |  |
| SHR |  | | |  | |  | |  | |  | |  |  | |  | |  |
| T1 | 1.00 (Reference) | | |  | |  | | 1.00 (Reference) | |  | |  | 1.00 (Reference) | |  | |  |
| T2 | 1.038 (0.832 ~ 1.295) | | | 0.742 | |  | | 1.044 (0.837 ~ 1.303) | | 0.701 | |  | 1.036 (0.826 ~ 1.299) | | 0.76 | |  |
| T3 | 1.603 (1.322 ~ 1.945) | | | <0.001 | |  | | 1.615 (1.331 ~ 1.96) | | <0.001 | |  | 1.539 (1.261 ~ 1.877) | | <0.001 | |  |
| P for trend | 1.295 (1.175 ~ 1.428) | | | <0.001 | |  | | 1.3 (1.179 ~ 1.433) | | <0.001 | |  | 1.265(1.145~ 1.398) | | <0.001 | |  |
| GV |  | | |  | |  | |  | |  | |  |  | |  | |  |
| T1 | 1.00 (Reference) | | |  | |  | | 1.00 (Reference) | |  | |  | 1.00 (Reference) | |  | |  |
| T2 | 1.56 (1.33 ~ 1.82) | | | <0.001 | |  | | 1.25 (1.07 ~ 1.47) | | 0.005 | |  | 1.02 (0.817 ~ 1.274) | | 0.86 | |  |
| T3 | 2.22 (1.91 ~ 2.58) | | | <0.001 | |  | | 1.60 (1.37 ~ 1.87) | | <0.001 | |  | 1.258 (0.988 ~ 1.602) | | 0.062 | |  |
| P for trend | 1.50 (1.43 ~ 1.56) | | | <0.001 | |  | | 1.48 (1.42 ~ 1.55) | | <0.001 | |  | 1.133 (1.004 ~ 1.279) | | 0.043 | |  |
| **Patients with NGR** |  | | |  | |  | |  | |  | |  |  | |  | |  |
| SHR |  | | |  | |  | |  | |  | |  |  | |  | |  |
| T1 | 1.00 (Reference) | | |  | |  | | 1.00 (Reference) | |  | |  | 1.00 (Reference) | |  | |  |
| T2 | 1.567 (0.926 ~ 2.653) | | | 0.094 | |  | | 1.542 (0.91 ~ 2.614) | | 0.108 | |  | 1.115 (0.849 ~ 2.515) | | 0.171 | |  |
| T3 | 2.32 (1.409 ~ 3.92) | | | 0.001 | |  | | 2.278 (1.382 ~ 3.755) | | 0.002 | |  | 2.05 (1.213 ~ 3.464) | | 0.007 | |  |
| P for trend | 1.508 (1.218 ~ 1.867) | | | <0.001 | |  | | 1.498 (1.209 ~ 1.856) | | 0.001 | |  | 1.422( 1.131~ 1.787) | | 0.003 | |  |
| GV |  | | |  | |  | |  | |  | |  |  | |  | |  |
| T1 | 1.00 (Reference) | | |  | |  | | 1.00 (Reference) | |  | |  | 1.00 (Reference) | |  | |  |
| T2 | 1.334 (0.95 ~ 1.875) | | | 0.097 | |  | | 1.323 (0.94 ~ 1.862) | | 0.108 | |  | 1.115 (0.772 ~ 1.609) | | 0.562 | |  |
| T3 | 1.93 (1.328 ~ 2.803) | | | 0.001 | |  | | 1.904 (1.306 ~ 2.777) | | 0.001 | |  | 1.766 (1.153 ~ 2.704) | | 0.009 | |  |
| P for trend | 1.388 (1.15 ~ 1.676) | | | 0.001 | |  | | 1.379 (1.14 ~ 1.668) | | 0.001 | |  | 1.321 (1.062 ~ 1.644) | | 0.013 | |  |
| **Patients with**  **Pre-DM** |  | | |  | |  | |  | |  | |  |  | |  | |  |
| SHR | | | |  | |  | |  | |  | |  |  | |  | |  |
| T1 | 1.00 (Reference) | | |  | |  | | 1.00 (Reference) | |  | |  | 1.00 (Reference) | |  | |  |
| T2 | 1.048 (0.704 ~ 1.559) | | | 0.818 | |  | | 1.068 (0.717 ~ 1.597) | | 0.745 | |  | 1.159 (0.751 ~ 1.788) | | 0.506 | |  |
| T3 | 1.341 (0.905 ~ 1.988) | | | 0.143 | |  | | 1.395 (0.939 ~ 2.073) | | 0.099 | |  | 1.557 (1 ~ 2.425) | | 0.05 | |  |
| P for trend | 1.162 (0.951 ~ 1.419) | | | 0.141 | |  | | 1.185 (0.969 ~ 1.449) | | 0.098 | |  | 1.25 ( 1 ~ 1.563) | | 0.05 | |  |
| GV |  | | |  | |  | |  | |  | |  |  | |  | |  |
| T1 | 1.00 (Reference) | | |  | |  | | 1.00 (Reference) | |  | |  | 1.00 (Reference) | |  | |  |
| T2 | 0.777 (0.519 ~ 1.163) | | | 0.219 | |  | | 0.768 (0.513 ~ 1.151) | | 0.201 | |  | 0.513 (0.323 ~ 0.815) | | 0.005 | |  |
| T3 | 1.455 (0.987 ~ 2.146) | | | 0.058 | |  | | 1.412 (0.915 ~ 2.09) | | 0.085 | |  | 0.993 (0.613 ~ 1.611) | | 0.978 | |  |

| **Variables** | **Model 1** | |  | **Model 2** | |  | **Model 3** | |
| --- | --- | --- | --- | --- | --- | --- | --- | --- |
|  |  |  |  |  |  |  |  |  |
|  | **HR (95%CI)** | **P** |  | **HR (95%CI)** | **P** |  | **HR (95%CI)** | **P** |
| P for trend | 1.227 (0.995 ~ 1.513) | 0.056 |  | 1.208 (0.978 ~ 1.492) | 0.08 |  | 1.037 (0.801 ~ 1.342) | 0.783 |
| **Patients with DM** |  |  |  |  |  |  |  |  |
| SHR |  |  |  |  |  |  |  |  |
| T1 | 1.00 (Reference) |  |  | 1.00 (Reference) |  |  | 1.00 (Reference) |  |
| T2 | 0.861 (0.607 ~ 1.221) | 0.4 |  | 0.857 (0.604 ~ 1.216) | 0.386 |  | 0.858 (0.595 ~ 1.237) | 0.413 |
| T3 | 1.651 (1.27 ~ 2.145) | <0.001 |  | 1.647 (1.267 ~ 2.141) | <0.001 |  | 1.7 (1.289 ~ 2.243) | <0.001 |
| P for trend | 1.317 (1.151 ~ 1.508) | <0.001 |  | 1.317 (1.15 ~ 1.508) | <0.001 |  | 1.335 ( 1.158~ 1.539) | <0.001 |
| GV |  |  |  |  |  |  |  |  |
| T1 | 1.00 (Reference) |  |  | 1.00 (Reference) |  |  | 1.00 (Reference) |  |
| T2 | 0.92 (0.632 ~ 1.397) | 0.758 |  | 0.96 (0.645 ~ 1.429) | 0.841 |  | 1.116 (0.732 ~ 1.702) | 0.609 |
| T3 | 0.822 (0.563 ~ 1.2) | 0.31 |  | 0.841 (0.574 ~ 1.231) | 0.373 |  | 0.997 (0.638 ~ 1.559) | 0.971 |
| P for trend | 1.123 (0.499 ~2.526) | 0.78 |  | 1.078 (0.945 ~ 1.228) | 0.264 |  | 0.968 (0.789 ~ 1.187) | 0.884 |

Table S1 (continued)

Table S2 Adjusted and Unadjusted Hazard Ratios of SHR, GV, and SHR×GV Interaction in NGR, Pre-DM, and DM Patients

| **Variables** | **Model 1** |  | **Model 2** | | |  | **Model 3** | |  |
| --- | --- | --- | --- | --- | --- | --- | --- | --- | --- |
|  | **HR (95%CI)** | **P** |  | **HR (95%CI)** | **P** |  | **HR (95%CI)** | **P** |  |
| **28-day mortality** |  |  |  |  |  |  |  |  |  |
| **Patients with NGR** | |  |  |  |  |  |  |  |  |
| SHR | 1.19 (0.76-1.87) | 0.436 |  | 1.15 (0.73-1.79) | 0.544 |  | 0.84 (0.53-1.33) | 0.463 |  |
| GV | 1.01 (0.58-1.76) | 0.975 |  | 0.93 (0.53-1.63) | 0.799 |  | 0.60 (0.33-1.09) | 0.092 |  |
| SHR×GV | 1.13 (0.90-1.41) | 0.301 |  | 1.17 (0.93-1.46) | 0.183 |  | 1.29 (1.02-1.64) | 0.033 |  |
| **Patients with Pre-DM** | |  |  |  |  |  |  |  |  |
| SHR | 2.76 (1.65-4.63) | <0.001 |  | 2.71 (1.61-4.55) | <0.001 |  | 2.31 (1.34-3.99) | 0.003 |  |
| GV | 2.96 (1.81-4.82) | <0.001 |  | 2.76 (1.69-4.52) | <0.001 |  | 2.23 (1.30-3.80) | 0.003 |  |
| SHR×GV | 0.71 (0.57-0.90) | 0.004 |  | 0.73 (0.58-0.92) | 0.007 |  | 0.75 (0.59-0.96) | 0.023 |  |
| **Patients with DM** |  |  |  |  |  |  |  |  |  |
| SHR | 2.10 (1.39-3.18) | <0.001 |  | 2.07 (1.36-3.14) | 0.001 |  | 1.76 (1.16-2.65) | 0.007 |  |
| GV | 1.43 (0.99-2.07) | 0.055 |  | 1.44 (1.00-2.08) | 0.053 |  | 1.31 (0.90-1.89) | 0.155 |  |
| SHR×GV | 0.85 (0.72-1.00) | 0.05 |  | 0.85 (0.72-1.00) | 0.05 |  | 0.85 (0.73-1.00) | 0.054 |  |
| **In ICU mortality** |  |  |  |  |  |  |  |  |  |
| **Patients with NGR** | | |  |  |  |  |  |  |  |
| SHR | | 0.86 (0.49-1.53) | 0.619 |  | 0.85 (0.48-1.52) | 0.585 |  | 0.86 (0.47-1.58) | 0.624 |
| GV | | 0.69 (0.33-1.42) | 0.314 |  | 0.68 (0.32-1.41) | 0.296 |  | 0.69 (0.32-1.49) | 0.339 |
| SHR×GV | | 1.32 (1.00-1.75) | 0.052 |  | 1.33 (1.00-1.77) | 0.05 |  | 1.30 (0.96-1.76) | 0.09 |
| **Patients with Pre-DM** | | |  |  |  |  |  |  |  |
| SHR | | 1.92 (1.05-3.53) | 0.035 |  | 1.84 (1.00-3.39) | 0.052 |  | 1.80 (0.94-3.46) | 0.077 |
| GV | | 1.99 (1.11-3.57) | 0.02 |  | 1.83 (1.02-3.31) | 0.044 |  | 1.47 (0.75-2.85) | 0.26 |
| SHR×GV | | 0.78 (0.59-1.02) | 0.066 |  | 0.80 (0.61-1.05) | 0.112 |  | 0.83 (0.62-1.12) | 0.226 |
| **Patients with DM** | |  |  |  |  |  |  |  |  |
| SHR | | 1.03 (0.61-1.73) | 0.912 |  | 1.04 (0.62-1.76) | 0.872 |  | 1.12 (0.66-1.90) | 0.674 |
| GV | | 0.72 (0.45-1.15) | 0.169 |  | 0.73 (0.46-1.17) | 0.195 |  | 0.84 (0.52-1.36) | 0.471 |
| SHR×GV | | 1.10 (0.90-1.35) | 0.335 |  | 1.10 (0.90-1.35) | 0.364 |  | 1.07 (0.87-1.31) | 0.515 |

Table S3 The results of the Proportional Hazards test

| Variable Name | p |
| --- | --- |
| SOFA | 0.000140379294574637 |
| GCS | 0.756409441038422 |
| Charlson | 8.27357309284934e-10 |
| HR | 0.194953510421609 |
| Abps | 7.17287048866999e-05 |
| Abpd | 0.00675309568771769 |
| Albumin | 0.121678317346374 |
| Lactate | 0.00310557922204439 |
| PH | 0.256505883800446 |
| Alt | 0.069331890965516 |
| Ast | 0.0178289534864053 |
| Creatinine | 0.0442201200667237 |
| BUN | 0.440475522541793 |
| Age | 1.00775064204004e-09 |
| BMI | 0.242527500965125 |
| Hemoglobin | 0.124469993180796 |
| Plt | 0.361941473277682 |
| RBC | 0.107801323087847 |
| WBC | 0.867135471371891 |
| HTN | 0.413030207319014 |
| AKI | 0.396605282494211 |
| HLD | 0.918564663057091 |
| HF | 0.0283241432189691 |
| COPD | 0.720646845075311 |
| Team | 0.024509946055759 |
| RI | 0.000550864077224185 |
| Hypoglycemic_drugs | 0.952143666363274 |
| Mechanical_ventilation | 0.49986369047985 |
| Sex | 0.488549416329834 |
| Temperature | 0.237080976553067 |
| Diabetes | 0.327717002631583 |
| GLOBAL | 1.8707203331523e-15 |

Table S4 The association of the combined SHR and GV indices with 28-day and ICU mortality in diabetic patients redefined by medical history and medication records

| **Variables** | **Model 1** |  | **Model 2** | | |  | **Model 3** | |
| --- | --- | --- | --- | --- | --- | --- | --- | --- |
|  | **HR (95%CI)** | **P** |  | **HR (95%CI)** | **P** |  | **HR (95%CI)** | **P** |
| **28-day mortality** |  |  |  |  |  |  |  |  |
| **Patients with DM** |  |  |  |  |  |  |  |  |
| Group 1 | 1.00 (Reference) |  |  | 1.00 (Reference) |  |  | 1.00 (Reference) |  |
| Group 2 | 1.54 (1.21 ~ 1.96) | <0.001 |  | 1.56 (1.23 ~ 1.99) | <0.001 |  | 1.17 (0.91 ~ 1.51) | 0.208 |
| Group 3 | 2.21 (1.67 ~ 2.93) | <0.001 |  | 2.22 (1.67 ~ 2.94) | <0.001 |  | 1.53 (1.14 ~ 2.05) | 0.004 |
| Group 4 | 2.42 (1.90 ~ 3.09) | <0.001 |  | 2.45 (1.91~ 3.12) | <0.001 |  | 1.36 ( 1.02~ 1.80) | 0.003 |
| P for trend | 1.34 (1.24 ~ 1.44) | <0.001 |  | 1.34 (1.25 ~ 1.45) | <0.001 |  | 1.15 (1.06 ~ 1.25) | 0.001 |
| **In ICU mortality** |  |  |  |  |  |  |  |  |
| **Patients with DM** |  |  |  |  |  |  |  |  |
| Group 1 | 1.00 (Reference) |  |  | 1.00 (Reference) |  |  | 1.00 (Reference) |  |
| Group 2 | 0.87 (0.65 ~ 1.17) | 0.355 |  | 0.88 (0.65 ~ 1.19) | 0.401 |  | 0.71 (0.52 ~ 0.96) | 0.029 |
| Group 3 | 1.13 (0.81 ~ 1.58) | 0.476 |  | 1.17 (0.83 ~ 1.64) | 0.364 |  | 1.04 (0.73 ~ 1.47) | 0.846 |
| Group 4 | 1.58 (1.18 ~ 2.11) | 0.002 |  | 1.63 (1.22 ~ 2.17) | 0.001 |  | 1.27 (0.94 ~ 1.72) | 0.130 |
| P for trend | 1.20 (1.09 ~ 1.32) | <0.001 |  | 1.21 (1.10 ~ 1.33) | <0.001 |  | 1.14 (1.03 ~ 1.26) | 0.009 |


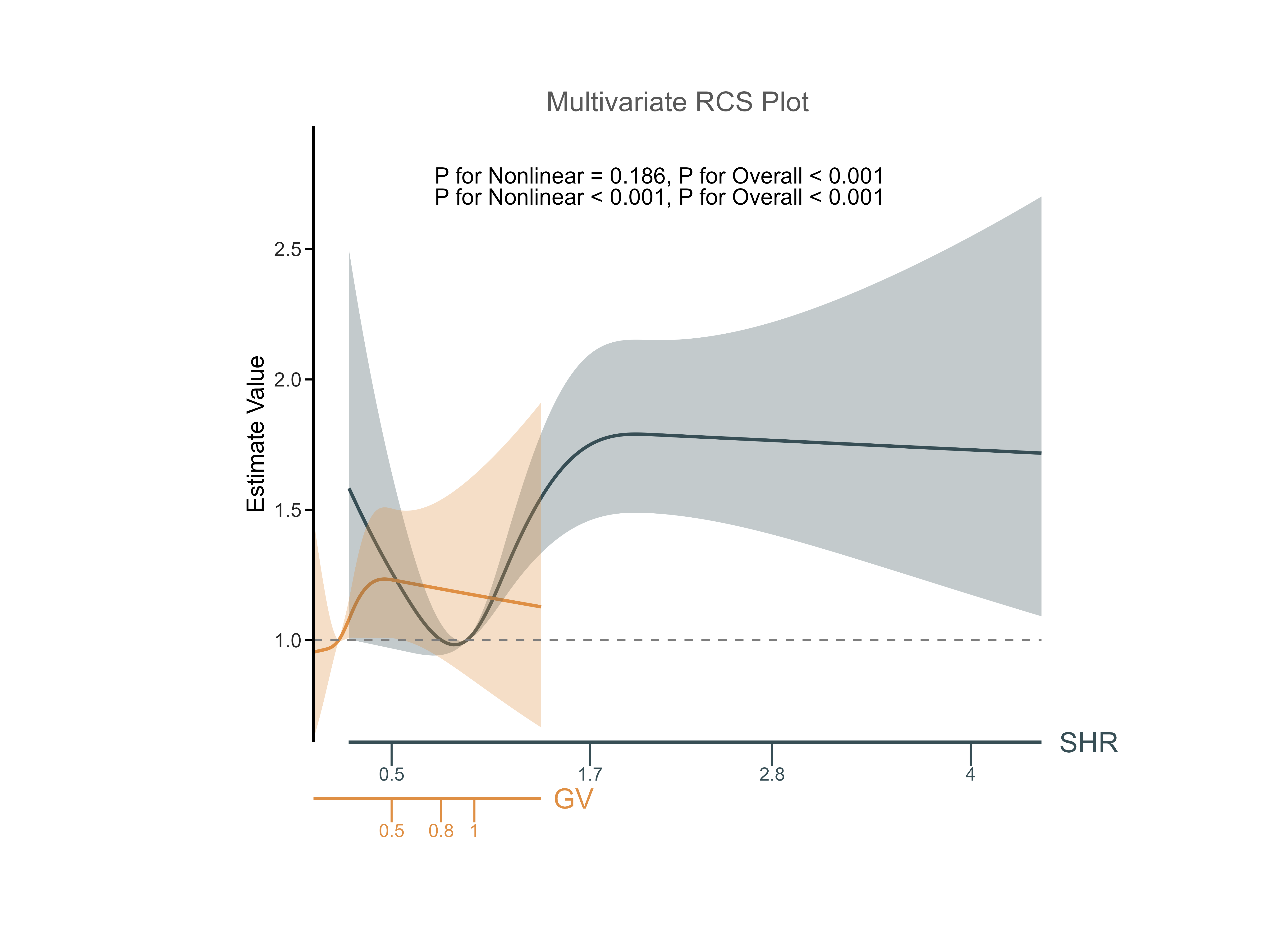

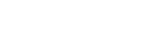


**Fig. S1** Multivariable-adjusted restricted cubic spline analyses of SHR and GV for 28-day mortality among all patients. SHR: P for nonlinear < 0.001, P for overall < 0.001; GV: P for nonlinear = 0.186, P for overall < 0.001


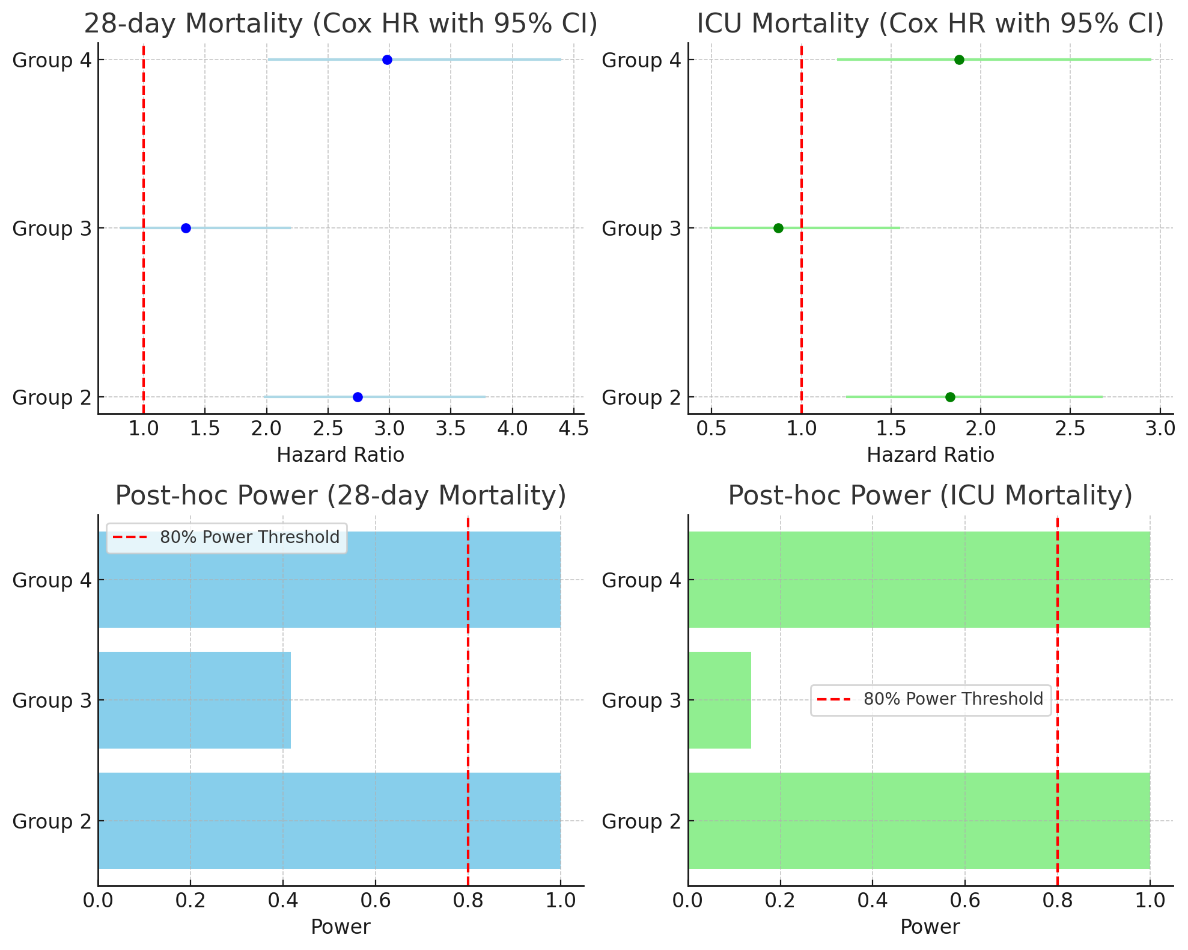


**Figure S2.** Hazard ratios (HRs) with 95% CIs and post-hoc power analyses for SHR/GV groups. Group 1: low SHR + low GV (reference), Group 2: high SHR + low GV, Group 3: low SHR + high GV, Group 4: high SHR + high GV.


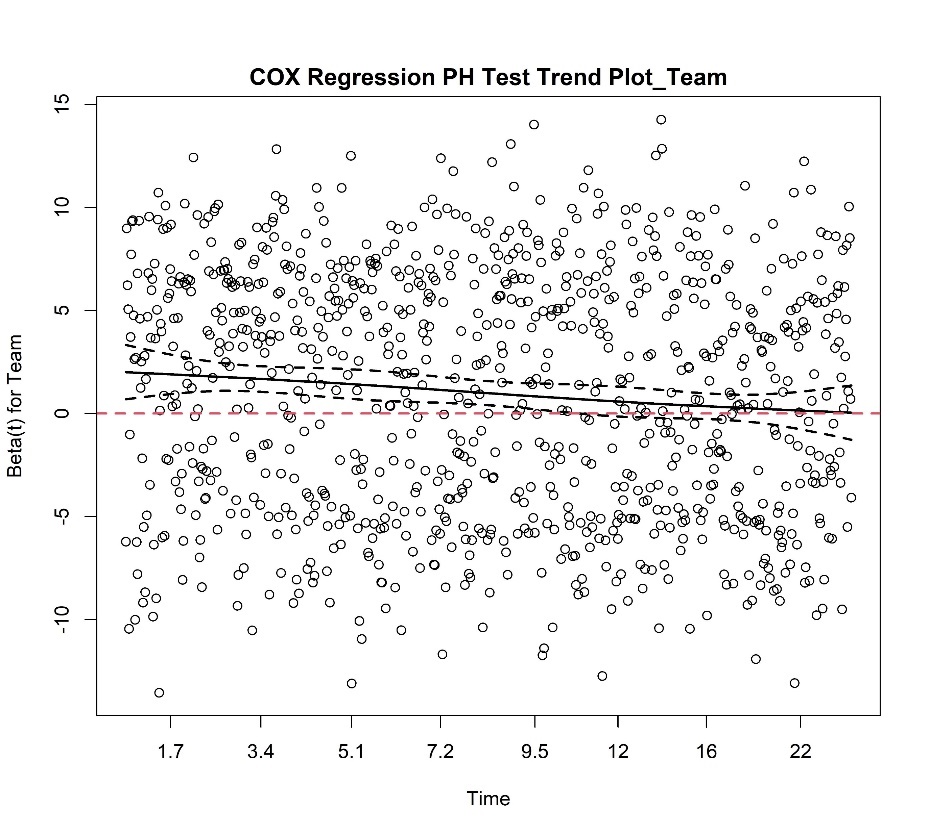


**Fig. S3** The trends of the proportional - hazards assumption test based on the COX regression model


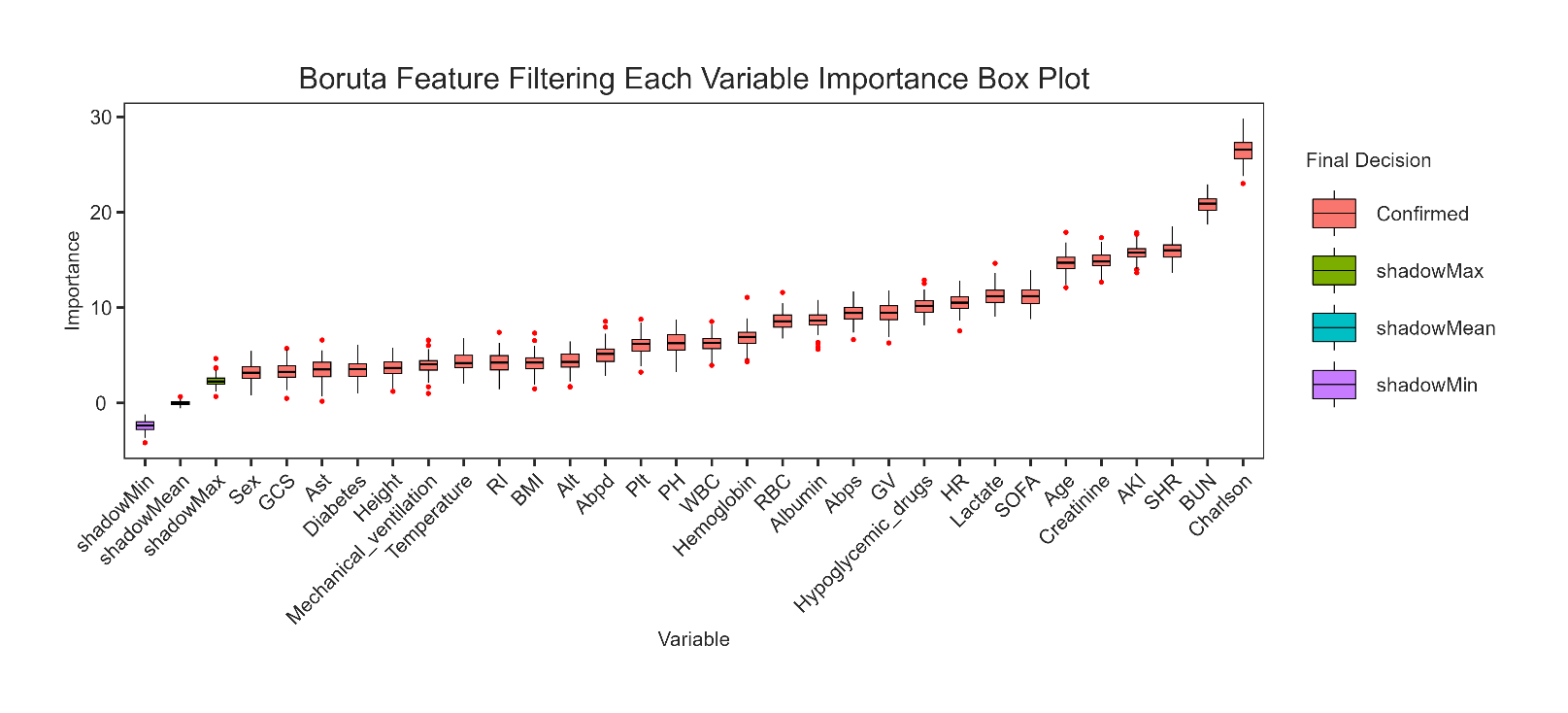


**Fig.S4** The Boruta algorithm ranks the importance of potential risk factors for 28-day mortality in overall patients.
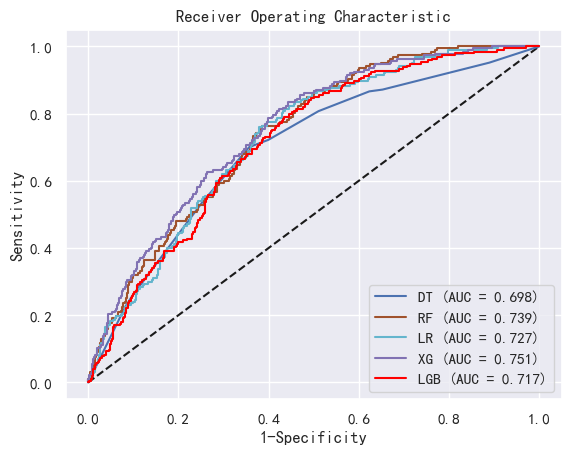
The horizontal axis represents the name of each variable, and the vertical axis represents the Z-value of each variable. The box plot shows the Z-values of each variable during the modelcalculation period.

**Fig.S5** Receiver operating characteristic curve of five ML models for predicting 28-day mortality in overall patients.


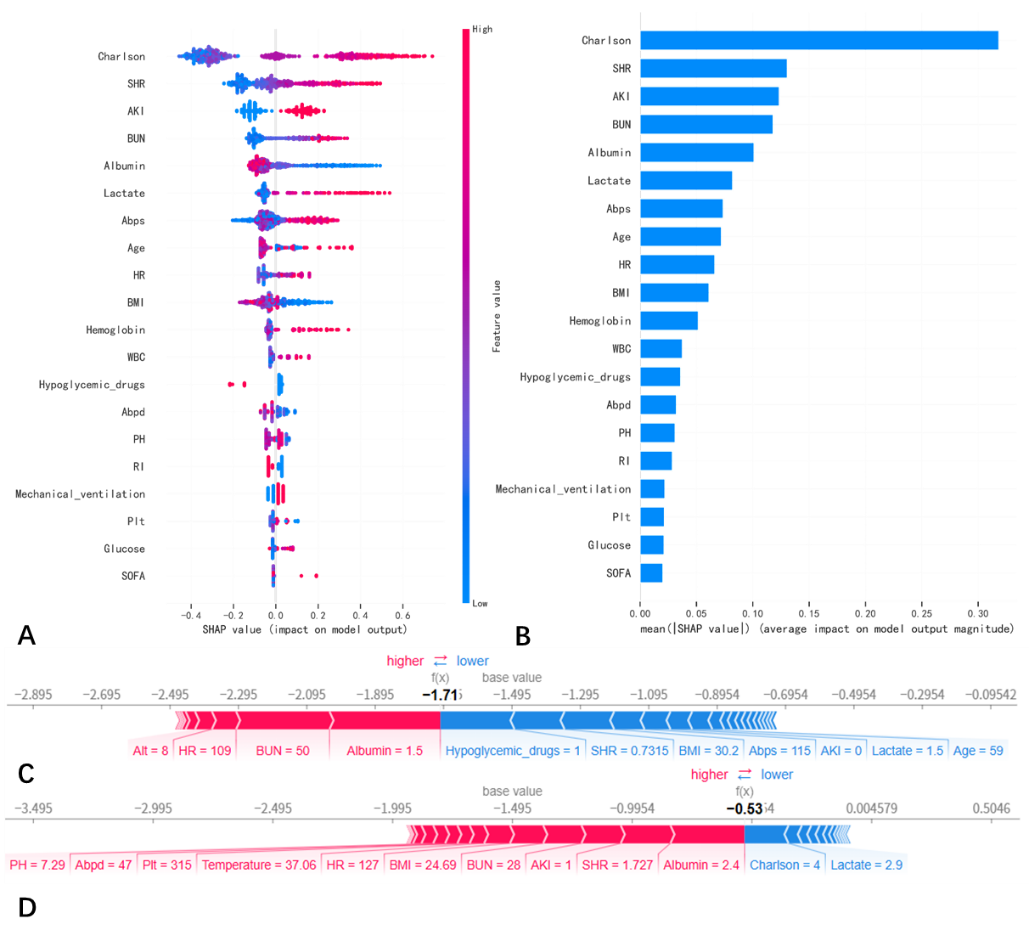


**Fig. S6** Explanation of the 28-day mortality prediction model in overall patients. (**A**), (**B**) Top 20 risk predictors for early prediction of 28-day mortality. Inference process of the model with (**C**) a surviving and (**D**) a non-surviving patient.


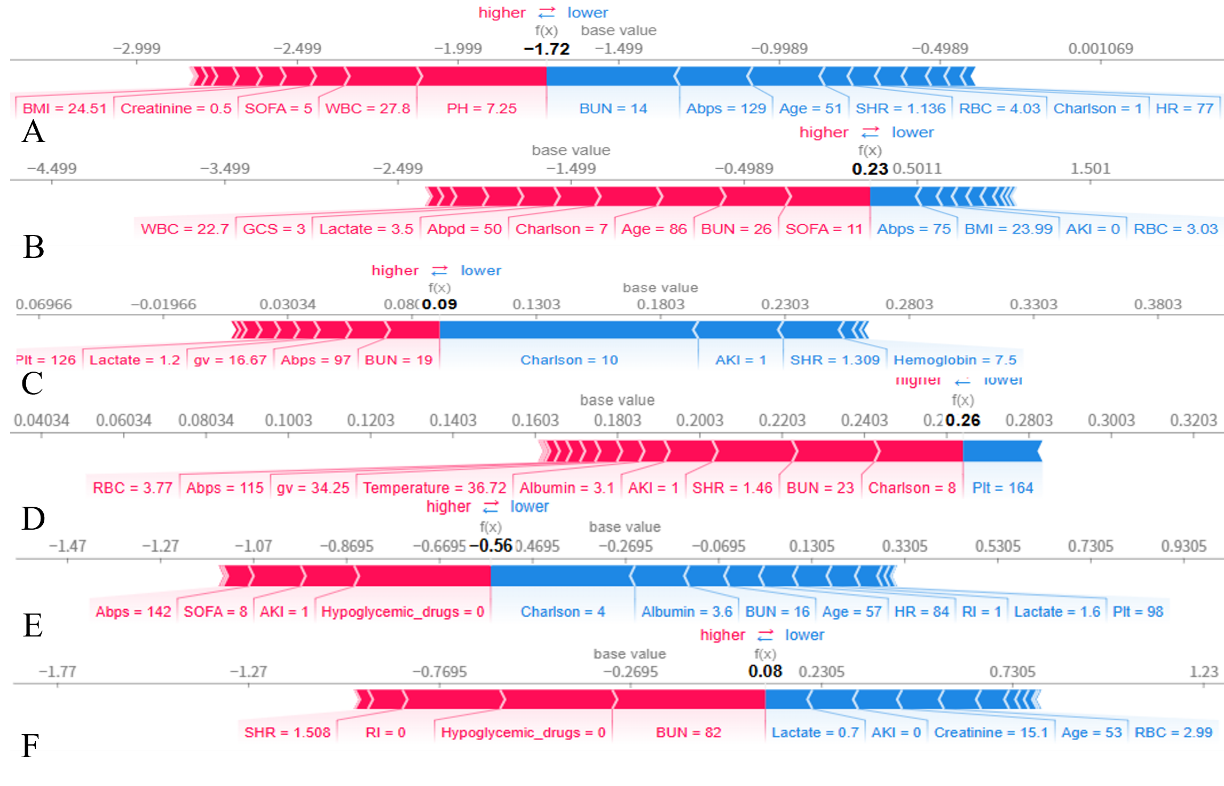


**Fig. S7** Inference process of the ML models with (**A, C, D**) surviving and (**B,D,F**) non-surviving patients.
